# Biomarker Discordances and Alterations Observed in Breast Cancer Treated with Neoadjuvant Chemotherapy: Causes, Frequencies, and Clinical Significances

**DOI:** 10.3390/curroncol29120761

**Published:** 2022-12-08

**Authors:** Cengiz Yilmaz, Demet Kocatepe Cavdar

**Affiliations:** 1 Department of Medical Oncology, Bozyaka Training and Research Hospital, The University of Health Sciences, Izmir 35170, Turkey; 2 Department of Pathology, Bozyaka Training and Research Hospital, The University of Health Sciences, Izmir 35170, Turkey

**Keywords:** breast cancer, neoadjuvant chemotherapy, biomarker discordance, biomarker alteration, chemosensitivity

## Abstract

Purpose: Biomarker discordances and alterations can be encountered between tru-cut biopsy and residual tumor in breast cancer treated with neoadjuvant chemotherapy (NACTx). We aimed to investigate the effect of NACTx on major biomarker expression (ER, PR, HER2, Ki-67) and tumor grade, the frequency and causes of receptor discordances, and the clinical significance of changes in terms of adjuvant therapy need and chemosensitivity. Methods: In this retrospective study, ER, PR, HER2, and Ki-67 expression and tumor grades were compared between pre- and post-NACTx tumor samples using the Wilcoxon signed-rank test. The frequencies of receptor discordances and the need for new adjuvant therapy due to discordances were calculated. The effect of patient and tumor characteristics and NACTx regimens on discordances was investigated using multivariate analysis. Using histopathological examinations, residual tumors were divided into chemotherapy-responsive and chemotherapy-unresponsive tumors. Biomarker changes in both groups were analyzed for predictability of chemosensitivity. Results: Of the 169 patients who received NACTx, 102 patients having enough residual tumors in the surgical pathology specimen were enrolled in the study. Histopathologically, about 70% of tumors were partially responsive to NACTx and 30% were unresponsive (chemo-resistant). The concordance and discordance rates were 95.1% versus 4.9% for ER (*p* = 0.180), 97.1% versus 2.9% for PR (*p* = 0.083), and 89.2% versus 10.8% for HER2 (*p* = 0.763), respectively. In addition, 15% of hormone receptor (HR)-negative patients became HR(+) and 5.7% of HER2(−) patients became HER2(+) in the residual tumors, requiring adjuvant endocrine or anti-HER2 therapy. In particular, 18% of triple-negative patients became HR(+) and 12% became HER2(+). HER2 loss was detected in 40% of HER2(+) patients. Multivariate logistic regression analysis revealed that lower estrogen expression (*p* = 0.046), a smaller tumor size (*p* = 0.029), and anti-HER2 therapy (*p* < 0.001) have independent efficacy on ER discordance, PR discordance, and HER2 discordance, respectively. Ki-67 and PR expression significantly decreased in chemotherapy-responsive tumors (*p* = 0.001 and *p* = 0.004), and the tumor grade increased in chemotherapy-unresponsive tumors (*p* = 0.034). Conclusions: Approximately 3–5% of HR discordance and about 10% of HER2 discordance can be observed in breast cancer after currently used NACTx regimens. Discordances are bi-directional (from positive to negative and vice versa), and their causes are multifactorial; they should be assessed accordingly. The NACTx effect alone cannot explain observed discordances but can cause biomarker alterations. The change in receptor status from positive to negative, especially HER2 loss, is mainly associated with the NACTx effect. However, the shift from negative to positive is thought to be primarily related to intratumoral heterogeneity. Receptor statuses becoming positive are of more clinical importance due to adjuvant therapy requirements. Biomarker alterations in PR, Ki-67, and tumor grade can provide predictive information about tumor chemosensitivity.

## 1. Introduction

Breast cancer is the most common type of cancer in women and is the leading cause of cancer-related deaths for women in most countries worldwide. The diagnosis and treatment of this cancer are an ongoing and highly challenging field for many researchers [1,2]. The pathological diagnosis of breast cancer is usually made by performing a tru-cut biopsy on a suspicious breast lesion [3,4]. The histological type, molecular subtype, and grade of tumors are determined using morphological examination and immunohistochemical (IHC) staining of the biopsy specimen. The molecular subtyping essential for assessing prognosis and planning treatment is established by analyzing major biomarkers, such as the estrogen receptor (ER), progesterone receptor (PR), human epidermal growth factor receptor-2 (HER2), and Ki-67 proliferation index [5,6]. For ER and PR positivity, IHC staining of at least 1% of tumor cells is recommended [7].

In locally advanced-stage and aggressive early-stage breast cancers, neoadjuvant chemotherapy (NACTx) is frequently used to allow breast-conserving surgery, reduce the risk of postoperative complications, and evaluate tumor chemosensitivity [8,9]. NACTx regimens containing anthracyclines and taxanes are generally used in the neoadjuvant treatment of breast cancer, and trastuzumab ± pertuzumab is added to the treatment in the case of HER2(+) disease [10,11].

Discordances in receptor statuses (from positive to negative or vice versa) and alterations in biomarker expressions can be observed between the tru-cut biopsies and post-NACTx residual tumors of patients without a pathological complete response (pCR). These inconsistencies are usually attributed to tumor heterogeneity, tissue processing errors, and pathological evaluations [12,13]. However, the effect of patient and tumor characteristics and the effect of currently used NACTx regimens on receptor discordances are not well known and should be investigated. There are some studies on post-NACTx receptor discordances in the literature. However, these studies are not fairly representative of the current situation, due to older NACTx regimens used and higher accepted cut-off values for ER and PR positivity [13,14].

Because a tru-cut biopsy may not reflect the actual tumor properties, due to intratumoral heterogeneity, receptor statuses in the tru-cut biopsy may be false negative. If the hormone receptor (HR) status becomes positive in a residual tumor, adjuvant endocrine therapy and if the HER2 status turns positive adjuvant trastuzumab therapy come into question. Since adjuvant endocrine and anti-HER2 therapies have been shown to prolong disease-free survival and overall survival, not administering adjuvant therapies in these situations may cause undertreatment [15,16,17,18]. Therefore, it seems critical to examine the receptor statuses of the residual tumor, especially if the receptor statuses are negative in the tru-cut biopsy. Knowing the percentage of HR(+) and HER2(+) patients missed because of negativity in the tru-cut biopsy will increase our clinical awareness of this critical issue.

In addition, it has been shown that pCR and the residual tumor burden are associated with disease-free survival and overall survival [19,20,21]. Consequently, the degree of pathological response correlates with survival and gives prognostic information. Similarly, the degree of changes in the tumor grade, Ki-67 proliferation index, and hormone expression levels of the tumors before and after NACTx may have prognostic value as in the case of the residual tumor burden in patients without pCR. Furthermore, investigating the biomarker alterations seen in chemo-sensitive and chemo-resistant tumors may increase our knowledge about chemosensitivity.

In this study, we aimed to investigate the frequency and causes of receptor discordances in breast cancer encountered after currently used NACTx regimens, their clinical significance in terms of adjuvant therapy requirements, the effect of NACTx on hormone expression levels, the Ki-67 proliferation index, and the tumor grade and to search for parameters that may indicate chemosensitivity by analyzing features of chemo-sensitive and chemo-resistant tumors.

## 2. Materials and Methods

### 2.1. Patient Selection

We retrospectively reviewed the medical records of 169 patients with breast cancer who received NACTx and underwent surgery in our center between January 2014 and September 2021. These patients’ pathology reports and previously stained surgical pathology slides were evaluated. Next, the patients were divided into four groups according to the degree of their tumor response to NACTx: those with pCR (no invasive tumor in the surgical pathology specimen), those with minimal residual disease (MRD; ≤10% residual invasive carcinoma), partially responsive patients, and unresponsive patients. Due to the absence of or an inadequate residual tumor, patients with pCR and MRD were excluded from the study. Those having enough residual tumors (partially responsive and unresponsive patients) were enrolled in the study.

### 2.2. Data Collection and Pathological Evaluations

Demographic data of the patients and their tumor characteristics, NACTx regimens used, and adjuvant therapies administered were obtained by reviewing the patients’ medical records. IHC results (ER, PR, and HER2 status; hormone expression levels; Ki-67 proliferation index) and other histopathological findings (histology and tumor grade) of the tru-cut biopsies and post-operative residual tumors were recorded from the original pathology reports of the patients. The ER and the PR status was accepted as positive if IHC staining of the corresponding receptor occurred in ≥1% of tumor cells. Any ER or PR positivity was considered HR(+) disease. A HER2 IHC score of 3 or a score of 2 with fluorescence in situ hybridization (FISH) positivity was considered HER2(+) disease. Triple negativity of ER, PR, and HER2 was considered triple-negative (TN) disease. Breast cancer was divided into five groups according to the molecular subtypes: luminal A (LA; ER strongly positive, PR ≥20% positive, HER2 negative, and Ki-67 proliferation index <14%), luminal B-HER2 negative (other HER2-negative luminal cancers), LB-HER2(+), HER2 enriched, and TN. Residual tumors were categorized as “partially responsive to NACTx” and “unresponsive to NACTx” by examining the patients’ pre-stained surgical pathology slides. Tumors that showed signs of regression on histopathological evaluation and also decreased pathological tumor size by ≥30% relative to the clinical tumor size or had at least a 30% reduction in tumor cellularity were considered “partially responsive to NACTx.” Those who did not exhibit signs of histopathological regression and did not have a sufficient decrease in tumor size or had increased tumor size were considered “pathologically unresponsive to NACTx.”

### 2.3. Data Evaluation and Statistical Analysis

The concordance and discordance rates of each receptor (ER, PR, HR, and HER2) were calculated by comparing the status of each receptor in tru-cut biopsies and residual tumors. Receptor status between tru-cut biopsy and residual tumor was considered concordant if it was the same and discordant if it was different. The impact of each receptor discordance on adjuvant therapy was determined. The effect of NACTx on hormone expression levels, tumor grade, and Ki-67 proliferation index was investigated by comparing the levels in the tru-cut biopsies and residual tumors. Partially responsive (chemo-sensitive) and unresponsive (chemo-resistant) tumor characteristics were analyzed to gain insight into chemosensitivity. So, changes in the levels of HR expression (ER% and PR%), tumor grade, and Ki-67 proliferation index were evaluated in both groups separately.

IBM^®^ SPSS^®^ statistics software version 21.0 was used for statistical analyses. The Wilcoxon signed-rank test was performed to compare pre-and post-treatment receptor statuses, hormone expression levels, tumor grades, and Ki-67 proliferation indices. Univariate and multivariate logistic regression analyses were performed to investigate the effect levels of factors on ER, PR, and HER2 discordances. A *p*-value of ≤0.05 was considered statistically significant.

**Figure 1:**
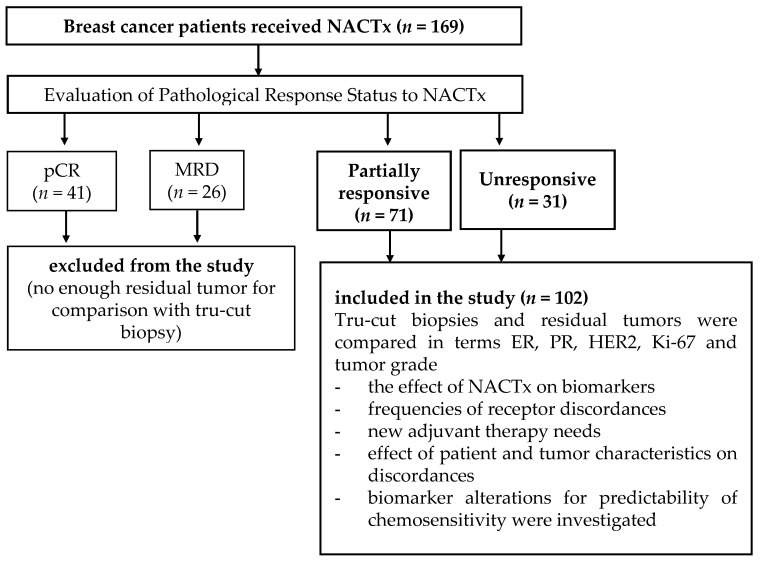
Study design, flow diagram, and purposes. NACTx: neoadjuvant chemotherapy; pCR: pathological complete response; MRD: minimal residual disease.

The study design and flow diagram are shown in Figure 1.

## 3. Results

### 3.1. Patients and Clinicopathological Characteristics

**Figure 2:**
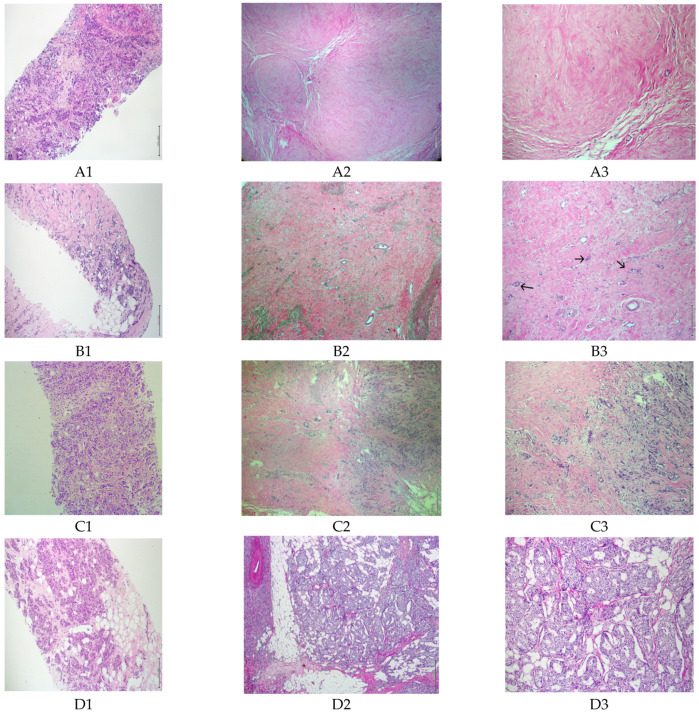
The study recruitment status of 169 patients with breast cancer according to the pathological response degree to NACTx. (**A1**) IBC on CNB, (×100, HE). (**A2**,**A3**) pCR (n = 41, excluded), microscopic appearance of the tumor bed after NACTx. Tumor bed with no residual tumor, characterized by stromal fibrosis and hyalinization, (×40 and ×100, HE). (**B1**) IBC on CNB, (×100, HE). (**B2**,**B3**) MRD with <10% of tumor remaining (n = 26, excluded). Stromal fibroelastosis and small clusters of residual invasive tumor cells (arrows), (×40 and ×100, HE). (**C1**) IBC on CNB, (×100, HE). (**C2**,**C3**) Partial response (n = 71, included). Some degree of fibrosis with residual tumor, (×40 and ×100, HE). (**D1**) IBC on CNB, (×100, HE). (**D2**,**D3**) No response to NACTx (n = 31, included), lumpectomy sample after NACTx, (×40 and ×100, HE). pCR: pathological complete response; MRD: minimal residual disease; NACTx: neoadjuvant chemotherapy; IBC: invasive breast carcinoma; CNB: core needle biopsy; HE: hematoxylin eosin.

Among the patients receiving NACTx for breast cancer (*n* = 169), 67 patients (39.6%) having pCR or near-pCR (MRD) were excluded from the study and 102 patients having enough residual tumors in the surgical pathology specimens were enrolled in the study (Figure 2). About 70% of these residual tumors were partially responsive to NACTx, and 30% were unresponsive to NACTx.

The mean age of the patients was 50.3 ± 9.6 years (range: 28–69). The distribution of the patients according to receptor statuses and molecular subtypes was as follows: 79.4% ER(+), 74.5% PR(+), 14.7% HER2(+),16% LA, 53% LB-HER2(−), 12% LB-HER2(+), 3% HER2 enriched, and 17% TN. Approximately 90% of the patients received NACTx containing anthracycline and taxane. Except for one, all HER2(+) patients received anti-HER2 drug(s). The clinicopathological features, NACTx regimens, and pathological response statuses of the patients are shown in Table 1.

### 3.2. Estrogen Receptor

Only 1 (1.2%) of the 81 ER(+) patients became ER(−), and 4 (19%) of the 21 ER(−) patients became ER(+) after NACTx. The total ER status change rate was 4.9% (5 patients; *p* = 0.180). The ER status did not change in 95.1% of the patients. The rate of effect of ER discordance on adjuvant therapy in ER(−) patients was 19% (4/21 patients; Table 2). The ER expression levels of four ER(−) patients who became ER(+) in the residual tumors were 70%, 10%, 10%, and 5%. The ER expression level of the single ER(+) patient who became ER(−) was 20%.

Significant efficacies of the tumor grade and pre-NACTx ER expression level were observed in predicting ER discordance in the univariate model of binary logistic regression analysis. However, in the multivariate model, only low levels of pre-NACTx ER expression had significant independent efficacy on ER discordance (OR: 0.940, 95% CI: 0.885–0.999, *p* = 0.046; Table 3).

### 3.3. Progesterone Receptor

Three (3.9%) of the 76 PR(+) patients became PR(−), and none (0%) of the 26 PR(−) patients became positive after NACTx. The total PR status change rate was 2.9% (3 patients; *p* = 0.083). The PR status did not change in 97.1% of the patients, and PR discordance did not affect adjuvant treatment (Table 2). The PR expression levels of the three PR(+) patients who became PR(−) in the residual tumors were 80%, 1%, and 1%.

In the univariate and multivariate logistic regression analysis model, only a small tumor size had significant independent efficacy on PR discordance (OR: 0.833, 95% CI: 0.707–0.987, *p* = 0.029; Table 4).

### 3.4. Hormone Receptor

No change was observed in 96.1% of the patients regarding their HR status. The HR status of four patients (3.9%) changed after NACTx (*p* = 0.317). One patient who was HR(+) in the tru-cut biopsy became HR(−) in the residual tumor. In contrast, 3 (15%) of the 20 patients who were HR(−) became HR(+) in the residual tumors after treatment. HR(−) patients who became HR(+) in their residual tumors were administered adjuvant endocrine therapy (tamoxifen or aromatase inhibitor; Table 2).

### 3.5. Human Epidermal Growth Factor Receptor-2

Of the 15 HER2(+) patients, 6 (40%) became HER2(−), and 5 (5.7%) of the 87 HER2(−) patients became HER2(+) in the residual tumors after NACTx. The total rate of change in the HER2 status was 10.8% (11 patients; *p* = 0.763). There was no change in the HER2 status in 89.2% of the patients. The rate of the effect of HER2 discordance on adjuvant therapy in HER2(−) patients was 5.7% (Table 2). Two of the five patients who became HER2(+) after NACTx had an IHC score of 3; the remaining three had an IHC score of 2 and were FISH(+). Adjuvant trastuzumab was administered to these patients. Of the six HER2(+) patients who became HER2(−) in the residual tumors, five had an IHC score of 3 and one had an IHC score of 2 and was FISH(+). Trastuzumab treatment was continued in these patients as well.

In the univariate binary logistic regression analysis model, significant efficacies of the pre-NACTx HER2 score (0–3), tumor grade, and anti-HER2 therapy were observed in predicting HER2 discordance. However, in the multivariate model, only administration of anti-HER2 therapy had significant independent efficacy on HER2 discordance (OR: 7.076, 95% CI: 2.437–20.542, *p* < 0.001; Table 5).

### 3.6. Molecular Subtype Changes

Furthermore, 1 of 16 LA patients and 2 of 54 LB-HER2(−) patients became HER2(+) in the residual tumors. Consequently, 4.3% of the patients with HER2(−) luminal tumors became HER2(+) postoperatively. Of the 17 TN patients, 2 patients (11.8%) became HER2(+) and 3 patients (17.6%) became HR(+) in the residual tumors. Of the 12 LB-HER2(+) patients, 50% became HER2(−). All three HER2-enriched patients remained the same postoperatively (Table 6).

### 3.7. Tumor Grade, Ki-67 Proliferation Index, and ER and PR Expression Levels

In general, no significant difference was observed in the mean tumor grade of patients before and after NACTx. However, a statistically significant decrease was observed in the mean Ki-67 proliferation index of the patients after NACTx (27.6 vs. 22.2, *p* = 0.001). A statistically insignificant increase was observed in the ER(+) cell percentage (62.4% vs. 66.1%, *p* = 0.062). In contrast, a statistically significant decrease was found in the PR(+) cell percentage after NACTx (51.2% vs. 42.3%, *p* = 0.001; Table 7).

### 3.8. Tumor Grade, Ki-67 Proliferation Index, and ER and PR Expression Levels of Partially Responsive and Unresponsive Patients

#### 3.8.1. Partially Responsive Patients

The mean tumor grade and ER expression level of these patients did not change significantly after NACTx. However, the mean Ki-67 proliferation index and PR expression level decreased significantly (mean Ki-67 proliferation index before and after NACTx: 27.5% vs. 20.6%, *p* = 0.001; mean PR expression level before and after NACTx: 49.9% vs. 40.5%, *p* = 0.004; Table 8).

#### 3.8.2. Unresponsive Patients

The mean ER and PR expression levels and Ki-67 proliferation index did not change significantly in these patients, whereas the mean tumor grade increased significantly (mean tumor grade before and after NACTx: 2.1 vs. 2.3, *p* = 0.034; Table 8).

## 4. Discussion

### 4.1. ER, PR, and HER2 Discordance without NACTx

Many studies have reported that there may be discordances in ER, PR, and HER2 statuses between tru-cut biopsy and surgical resection material in patients with breast cancer who have not undergone NACTx. Concordance rates were between 62% and 99% for ER, 69% and 89% for PR, and 54% and 100% for HER2 [6,22,23,24,25,26,27,28,29,30,31,32]. Seferina et al. reported the concordance rates as 89.5% for ER, 82.5% for PR, and 80.6% for HER2. The false-negative rates in their study were 26.5% for ER, 29.6% for PR, and 5.4% for HER2 [6]. If the overall rate of HR(+) breast cancer is assumed to be 70%, approximately 20–30% of the remaining 30% HR(−) patients will be mistakenly considered HR(−) based on tru-cut biopsy results, even though they are HR(+). Therefore, approximately 5–10% of the patients may be deprived of adjuvant endocrine therapy. Similarly, if the rate of HER2(+) breast cancer is accepted as 20%, 5.4% of the remaining 80% patient group will be considered HER2(−) according to tru-cut biopsy results, even though they are HER2(+), and approximately 4–5% of these patients will be devoid of anti-HER2 treatments. Consequently, receptor discordances, which may have clinical significance in terms of adjuvant therapy, can frequently be observed even in patients with breast cancer who have not been treated with NACTx.

### 4.2. ER, PR, and HER2 Discordance after NACTx

Van de Ven et al. reported that chemotherapy affects tumor biology directly or indirectly and causes receptor discordance in breast cancer. They evaluated studies conducted between 1996 and 2009 on receptor discordance after NACTx. The ER discordance rate was between 2.5% and 17% in eight studies, and there was no discordance in seven studies; the PR discordance rate was between 5.9% and 51.7% in four studies, and there was no discordance in five studies [13]. However, the cut-off values for ER and PR positivity in these studies were accepted as 5% or 10%. In addition, patients were generally administered only anthracycline-based and taxane-free NACTx regimens, which are rarely used today. In our study, the cut-off value for ER and PR positivity was accepted as ≥1%, according to the recommendations of the American Society of Clinical Oncology [7]. In addition, sequential NACTx regimens containing anthracycline and taxane, which are commonly used today, were generally administered, and anti-HER2-targeted drug(s) were added to the treatment in the case of HER2(+) disease [33]. In the same meta-analysis, 19 studies were examined for HER2 status and only 3 studies that used trastuzumab in addition to NACTx were evaluated. According to the results of these three studies, 12%–43% of the patients who were HER2(+) before treatment became HER2(−) in the post-treatment residual disease, while none of the HER2(−) patients became HER2(+) [34,35,36]. According to the results of our study, 40% of the HER2(+) patients lost their HER2 status. Unlike previous studies, 5.7% of HER2(−) patients became HER2(+). Two of these patients had an IHC score of 3, and three had an IHC score of 2 and were FISH(+). The fact that FISH was not performed in all patients is a limitation of our study. As a result, our study is significantly distinct from previous studies because of the currently used NACTx regimens and up-to-date cut-off values for ER and PR. According to the results of our research, which is believed to represent the current situation more accurately, the concordance and discordance rates between the tru-cut biopsy before NACTx and the residual tumor after NACTx were 95.1% vs. 4.9% for ER, 97.1% vs. 2.9% for PR, 96.1% vs. 3.9% for HR, and 89.2% vs. 10.8% for HER2, respectively.

The effect of NACTx on discordances in ER, PR, HR, and HER2 statuses between the tru-cut biopsy and the residual tumor was analyzed in our study. NACTx alone could not explain observed discordances (*p*-values: 0.18, 0.08, 0.32, and 0.76, respectively). The tru-cut biopsy specimen is usually a tiny fraction of the entire tumor and may not represent actual tumor properties, due to intratumoral heterogeneity. In addition, the tru-cut procedure and the experience of interventional radiologists, sampling errors, tissue preparation, fixation problems, quality of IHC stains, and interpretation differences among pathologists may cause discordances [26,37,38]. However, the impact of NACTx still cannot be ignored. The high rate of HER2 status loss after NACTx in HER2(+) patients and the statistically significant decrease in PR expression levels in partially responsive patients can be explained by the effect of NACTx. Logistic regression analysis also revealed the influence of anti-HER2 treatments on HER2 discordance, supporting the impact of chemotherapy on discordance. Probably, HER2(–) patients becoming HER2(+) in the residual tumors due to tumor heterogeneity neutralizes the HER2 loss effect of NACTx and prevents the statistical significance of NACTx on HER2 discordance (10.8%; *p* = 0.76). Since the discordance is bidirectional (from positive to negative and from negative to positive), it would be more appropriate to investigate the effects of factors on each side of the discordance separately with a higher number of patients. As a result, discordances are caused by a combination of tumor heterogeneity, the NACTx effect, and other factors. If the influence of other factors is ignored, the change in receptor status from positive to negative can be explained mainly by the effect of NACTx. However, the shift from negative to positive can be explained by intratumoral heterogeneity rather than chemotherapy.

### 4.3. Discordance with Clinical Significance

Discordances in HRs, HER2, tumor grade, and Ki-67 proliferation index can have several clinical significances regarding prognosis, adjuvant treatment, and chemosensitivity or chemoresistance. In terms of prognosis, Ozdemir et al. reported that patients having ER- or PR-positive tumors before NACTx and becoming negative in the residual tumors have shorter overall survival than those with an unchanged positive HR status [39]. However, a receptor status that is negative in the tru-cut biopsy but positive in the residual tumor is challenging and may affect the decision for adjuvant therapy. The adjuvant treatment decision is usually made according to the receptor statuses in the tru-cut biopsy performed before NACTx. Patients who are HR(+), according to tru-cut biopsy results, are administered adjuvant endocrine therapy regardless of the HR status in the residual tumor. Similarly, trastuzumab is administered during the neoadjuvant and adjuvant periods in HER2(+) disease. Adjuvant hormonal therapy and/or trastuzumab should be initiated in patients who are HR(−) and/or HER2(−) in the tru-cut biopsy and become positive in the residual tumor after treatment. In our study, the receptor status of 15% of HR(−) patients and 5.7% of HER2(−) patients changed from negative to positive. These patients were administered adjuvant endocrine and/or trastuzumab therapy. It would be more appropriate to initiate adjuvant trastuzumab therapy after confirmation with a FISH test for patients who are HER2(−) in the tru-cut biopsy and become HER2(+) in the residual tumor. Discordances also may provide clinical information about chemosensitivity, as discussed in Section 4.5.

### 4.4. Molecular Subtype Changes

Some LA tumors appeared to differentiate into more aggressive subtypes after NACTx, such as LB-HER2(−) at a rate of 37.5% and LB-HER2(+) at a rate of 6.3%. In addition, two LB-HER2(−) tumors (3.7%) became HER2(+) and one (1.9%) differentiated into the TN molecular subtype. Of the TN patients, 11.8% became HER2(+) and 17.7% became HR(+). However, 50% of the LB-HER2(+) patients lost their HER2 status. Under NACTx, which usually comprises anthracyclines and taxanes, it is not expected that LA tumors will differentiate into other aggressive molecular subtypes, LB-HER2(−) tumors will acquire HER2(+) status, or TN tumors will become HR(+) or HER2(+). This discordance, which turned positive from negative, can be mainly explained because the tru-cut biopsy does not reflect the entire tumor structure. However, the conversion of LB-HER2(+) tumors to HER2(−) tumors can be easily explained by NACTx containing anti-HER2-targeted therapy that also showed statistically significant efficacy in multivariate logistic regression analysis.

### 4.5. Changes in ER and PR Expression Levels, Tumor Grade, and Ki-67 Proliferation Index before and after NACTx

In the meta-analysis of Van de Ven et al., 10 studies were evaluated for ER expression, and it was reported that the expression level changed in 4 and remained unchanged in 6 of these studies [13]. PR expression decreased considerably in four of the seven studies evaluated. In our study, the mean ER expression levels increased after NACTx, although the increase was not statistically significant. However, post-NACTx PR expression levels decreased considerably. Our study differs from previous studies because patients with and without pathological responses were evaluated separately. PR expression levels significantly reduced in pathologically responsive patients. Although they decreased in pathologically unresponsive patients, the decrease was not statistically significant. When considered together with previous studies, the decrease in the level of PR expression can be a marker for chemosensitivity.

There was no significant change in the mean tumor grade of all study patients. However, the mean tumor grade of unresponsive patients was considerably higher after NACTx. An increased tumor grade under NACTx requires investigating aggressive molecular subtypes with high tumor grades unaffected by chemotherapy. It has been reported that tumors with a high Ki-67 proliferation index are more likely to respond to NACTx and have a higher pCR rate [40]. In our study, no difference was noted between the mean pre-treatment Ki-67 proliferation indices of patients with and without a pathological response (27.5 vs. 27.8). Since patients with pCR and MRD were not included in our study, it may not be appropriate to conclude on this issue. However, there was a statistically significant decrease in the mean Ki-67 proliferation index of tumors pathologically responding to chemotherapy, whereas there was no substantial change in tumors not responding to chemotherapy. Consequently, a decreased Ki-67 proliferation index and PR expression level may indicate chemosensitivity in patients with breast cancer treated with NACTx. An increase in tumor grade may be an indicator of chemoresistance.

## 5. Limitations

The results of our study should be considered in the light of some limitations. Two major limitations are that the study is not prospective and the number of patients is not high. Since this is a retrospective study, biomarkers were examined by different physicians in the long term under varying conditions; therefore, evaluations were not standardized. In addition, some other factors other than histopathological evaluation, such as tissue-processing errors and stains that were used, may have affected biomarker statuses. Powerful and more accurate statistical analyses can be performed with a larger number of patients. Another limitation of the study is that all patients with HER2-positive residual tumors were not confirmed with a FISH test.

## 6. Conclusions

Receptor statuses may change after NACTx in patients with breast cancer. Approximately 3–5% discordance in the HR status and about 10% discordance in the HER2 status can be observed in breast cancer treated with NACTx regimens commonly used today. The NACTx effect alone cannot explain observed discordances. The change in receptor status from positive to negative, especially HER2 loss, is mainly associated with the NACTx effect. However, the shift from negative to positive is thought to be primarily related to intratumoral heterogeneity. Because discordances are bidirectional (from positive to negative and from negative to positive) and etiologies are multifactorial, further studies or meta-analyses should be conducted separately according to both aspects of discordance to obtain more conclusive results. Receptor statuses becoming positive are of more clinical importance due to adjuvant therapy requirements. Therefore, IHC staining should be repeated and the residual tumor’s receptor statuses should be examined. The status of patients who are HER2(−) in the tru-cut biopsy and have a HER2 IHC score of 2 or 3 in the residual tumors should be confirmed using a FISH test. After treatment, an increase in the tumor grade may indicate chemotherapy resistance, whereas a decrease in the PR expression level and Ki-67 proliferation index may indicate chemosensitivity. Well-designed prospective studies with a large number of patients should be performed to reveal alterations associated with chemosensitivity and the factors affecting receptor discordances, definitely.

## Figures and Tables

**Table 1:** Clinicopathological features, NACTx regimens, and pathological outcomes of the patients.

Variables	Mean or Subgroups	n (%)
Age, years	Mean ± SD (range)	50.3 ± 9.6 (28–69)
Menopausal status	Pre-menopausal	45 (44.1)
	Peri-menopausal	8 (7.8)
	Post-menopausal	49 (48.0)
Clinical stage	Early	54 (52.9)
	Locally advanced	37 (36.3)
	Inflammatory	5 (4.9)
	Oligo-metastatic	6 (5.9)
Tumor size, mm	Mean ± SD (range)	29 ± 13 (5–85)
cT	T1	11 (10.8)
	T2	68 (66.7)
	T3	4 (3.9)
	T4	19 (18.6)
cN	N0	6 (5.9)
	N1	66 (64.7)
	N2	26 (25.5)
	N3	4 (3.9)
Histology	IDC	90 (88.2)
	ILC	6 (5.9)
	Mixed	2 (2.0)
	Other	4 (3.9)
Centricity	Unicentric	90 (88.2)
	Multicentric	12 (11.8)
Solitary tumor vs. multiple tumors	Solitary	45 (44.1)
	Multiple	57 (55.9)
Molecular subtype	Luminal A	16 (15.7)
	LB-HER2(−)	54 (52.9)
	LB-HER2(+)	12 (11.8)
	HER2 enriched	3 (2.9)
	Triple negative	17 (16.7)
NACTx regimen	EC-w paclitaxel	32 (31.4)
	DD AC-w paclitaxel	39 (38.2)
	DD AC-docetaxel	14 (13.7)
	AC-taxane	6 (5.9)
	FEC-docetaxel	1 (1.0)
	Only anthracycline based	6 (5.9)
	Only taxane based	4 (3.9)
Anti-HER2 drug(s)	Not received	88 (86.3)
	Trastuzumab	10 (9.8)
	Trastuzumab + pertuzumab	4 (3.9)
Pathological response status	Partially responsive to NACTx	71 (69.6)
	Unresponsive to NACTx	31 (30.4)

NACTx: neoadjuvant chemotherapy; cT: clinical tumor stage; cN: clinical lymph node stage; IDC: invasive ductal carcinoma; ILC: invasive lobular carcinoma; LB: luminal B; EC: epirubicin + cyclophosphamide; w: weekly; DD: dose-dense; AC: adriamycin + cyclophosphamide; FEC: fluorouracil + epirubicin + cyclophosphamide.

**Table 2:** ER, PR, HR, and HER2 status of the patients before and after NACTx and clinical outcomes.

Tru-Cut Bx	Post-NACTx Residual Tumor, *n* (%)		Total *n* (%)	Concordance	Discordance	*p*-Value ^w^	Clinical Outcome	
	Positive	Negative					Positive to (−)	Negative to (+)
ER Status				95.1%	4.9%	0.180	1.2%	19%
ER(+)	80 (98.8)	1 (1.2)	81 (79.4)				Adjuvant ET	Adjuvant ET
ER(−)	4 (19.0)	17 (81.0)	21 (20.6)					
Total	84 (82.4)	18 (17.6)	102 (100.0)					
PR Status				97.1%	2.9%	0.083	3.9%	0%
PR(+)	73 (96.1)	3 (3.9)	76 (74.5)				Adjuvant ET	Adjuvant ET
PR(−)	0 (0.0)	26 (100.0)	26 (25.5)					
Total	73 (71.6)	29 (28.4)	102 (100.0)					
HR Status				96.1%	3.9%	0.317	1.2%	15% *
HR(+)	81 (98.8)	1 (1.2)	82 (80.4)				Adjuvant ET	Adjuvant ET
HR(−)	3 (15.0) *	17 (85.0)	20 (19.6)					
Total	84 (82.4)	18 (17.6)	102 (100.0)					
HER2 Status				89.2%	10.8%	0.763	40%	5.7% *
HER2(+)	9 (60.0)	6 (40.0)	15 (14.7)				Adjuvant Trastuzumab	Adjuvant Trastuzumab
HER2(−)	5 (5.7) *	82 (94.3)	87 (85.3)					
Total	14 (13.7)	88 (86.3)	102 (100.0)					

ER: estrogen receptor; PR: progesterone receptor; HR: hormone receptor; NACTx: neoadjuvant chemotherapy; Bx: biopsy; ET: endocrine treatment; * additional patients requiring adjuvant therapy after NACTx; ^w^ Wilcoxon signed-rank test.

**Table 3:** Binary logistic regression analysis for ER discordance.

ER Discordance	Univariate Model					Multivariate Model				
	OR	95% CI			*p*-Value	OR	95% CI			*p*-Value
Age (years)	0.934	0.847	-	1.029	0.168					
Tumor size (mm)	0.995	0.928	-	1.067	0.886					
Pre-NACTx ER expression (%)	0.940	0.885	-	0.999	0.046	0.940	0.885	-	0.999	0.046
Pre-NACTx Ki-67 proliferation index (%)	1.039	0.996	-	1.084	0.075					
Pre-NACTx tumor grade	9.584	1.057	-	86.896	0.045					0.292
NACTx regimen	1.079	0.640	-	1.818	0.776					
Histology	-	-	-	-	0.999					
Centricity (unicentric vs. multicentric)	-	-	-	-	0.999					
Solitary tumor vs. multiple tumors	0.837	0.134	-	5.237	0.849					
Clinical tumor stage	0.614	0.177	-	2.130	0.614					
Clinical lymph node stage	1.370	0.355	-	5.297	0.648					

Logistic regression (forward LR).

**Table 4:** Binary logistic regression analysis for PR discordance.

PR Discordance	Univariate Model					Multivariate Model				
	OR	95% CI			*p*-Value	OR	95% CI			*p*-Value
Age (years)	1.093	0.952	-	1.254	0.209					
Tumor size (mm)	0.833	0.707	-	0.982	0.029	0.833	0.707	-	0.982	0.029
Pre-NACTx PR expression (%)	0.984	0.953	-	1.016	0.317					
Pre-NACTx Ki-67 proliferation index (%)	1.033	0.980	-	1.090	0.230					
Pre-NACTx tumor grade (1–3)	1.377	0.177	-	10.693	0.760					
NACTx regimen	0.813	0.323	-	2.048	0.661					
Histology	-	-	-	-	0.998					
Centricity (unicentric vs. multicentric)	4.000	0.335	-	47.810	0.273					
Solitary tumor vs. multiple tumors	1.600	0.140	-	18.228	0.705					
Clinical tumor stage	2.994	0.876	-	10.230	0.080					
Clinical lymph node stage	0.437	0.054	-	3.548	0.437					

Logistic regression (forward LR).

**Table 5:** Binary logistic regression analysis for HER2 discordance.

HER2 Discordance	Univariate Model					Multivariate Model				
	OR	95% CI			*p*-Value	OR	95% CI			*p*-Value
Age (years)	1.053	0.985	-	1.129	0.147					
Tumor size (mm)	1.002	0.957	-	1.049	0.924					
Pre-NACTx HER2 score (0–3)	3.732	1.782	-	7.818	<0.001					0.806
Pre-NACTx Ki-67 proliferation index (%)	1.012	0.979	-	1.046	0.467					
Pre-NACTx tumor grade (1–3)	4.683	1.322	-	16.592	0.017					0.146
NACTx regimen	0.747	0.436	-	1.281	0.289					
Anti-HER2 therapy	7.076	2.437	-	20.542	<0.001	7.076	2.437	-	20.542	<0.001
Histology	0.629	0.139	-	2.840	0.547					
Centricity (unicentric vs. multicentric)	0.727	0.085	-	6.244	0.772					
Solitary tumor vs. multiple tumors	4.031	0.825	-	19.699	0.085					
Clinical tumor stage	1.085	0.548	-	2.147	0.815					
Clinical lymph node stage	0.995	0.367	-	2.694	0.992					

Logistic regression (forward LR).

**Table 6:** Molecular subtypes of the patients before and after NACTx.

Tru-Cut Bx	Post- NACTx Residual Tumor					Total, *n* (%)
Molecular Subtypes	LA	LB-HER2(−)	LB-HER2(+)	HER2 Enriched	TN	
LA, n (%)	9 (56.3)	6 (37.5)	1 (6.3)	0 (0.0)	0 (0.0)	16 (100.0)
LB-HER2(−), n (%)	19 (35.2)	32 (59.3)	2 (3.7)	0 (0.0)	1 (1.9)	54 (100.0)
LB-HER2(+), n (%)	1 (8.3)	5 (41.7)	6 (50.0)	0 (0.0)	0 (0.0)	12 (100.0)
HER2 enriched, n (%)	0 (0.0)	0 (0.0)	0 (0.0)	3 (100.0)	0 (0.0)	3 (100.0)
TN, n (%)	0 (0.0)	2 (11.8)	1 (5.9)	1 (5.9)	13 (76.5)	17 (100.0)
Total, *n* (%)	29 (28.4)	45 (44.1)	10 (9.8)	4 (3.9)	14 (13.7)	102 (100.0)

NACTx: neoadjuvant chemotherapy; Bx: biopsy; LA: luminal A; LB: luminal B; TN: triple negative.

**Table 7:** Tumor grade, Ki-67 proliferation index, and ER and PR expression levels before and after NACTx.

Category	Tru-Cut	Post-op	Stable (*n*)	Decrease (*n*)	Increase (*n*)	*p*-Value ^w^
Grade, mean	2.24 ± 0.6	2.25 ± 0.6	70	15	17	0.724
Ki-67 proliferation index, mean %	27.6 ± 17.5	22.2 ± 18.7	13	59	30	0.001
ER, mean %	62.4 ± 36.2	66.1 ± 37.4	47	20	35	0.062
PR, mean %	51.2 ± 39.9	42.3 ± 37.0	40	44	18	0.001

NACTx: neoadjuvant chemotherapy; ER: estrogen receptor; PR: progesterone receptor; post-op: post-operative; ^w^ Wilcoxon signed-rank test.

**Table 8:** Tumor grade, Ki-67 proliferation index, and ER and PR expression levels of partially responsive and unresponsive patients.

Category		Partially Responsive (*n* = 71)	*p*-Value ^w^	Unresponsive (*n* = 31)	*p*-Value ^w^
Grade, mean	Tru-cut	2.3 ± 0.5	0.414	2.1 ± 0.61	0.034
	Post-op	2.2 ± 0.6		2.3 ± 0.6	
Ki-67 proliferation index, mean %	Tru-cut	27.5 ± 15.8	0.001	27.8 ± 21.1	0.446
	Post-op	20.6 ± 15.1		26.1 ± 25.1	
ER, mean %	Tru-cut	64.1 ± 35.8	0.266	58.9 ± 37.5	0.124
	Post-op	67.2 ± 35.9		63.5 ± 41.1	
PR, mean %	Tru-cut	49.9 ± 40.6	0.004	54.2 ± 38.8	0.132
	Post-op	40.5 ± 36.3		46.4 ± 38.7	

NACTx: neoadjuvant chemotherapy; ER: estrogen receptor; PR: progesterone receptor; post-op: post-operative; ^w^ Wilcoxon signed-rank test.

## Data Availability

The data presented in this study are available on request from the corresponding author.

## Abbreviations

ER	Estrogen receptor
PR	Progesterone receptor
HR	Hormone receptor
LA	Luminal A
FISH	Fluorescence in situ hybridization
pCR	Pathological complete response
NACTx	Neoadjuvant chemotherapy
TN	Triple negative
HER2	Human epidermal growth factor receptor 2
IHC	Immunohistochemical staining
MRD	Minimal residual disease
